# Cognitive Performances: The Role of Digit Ratio (D2:D4) With a Protective Factor for Anxiety

**DOI:** 10.3389/fnrgo.2022.870362

**Published:** 2022-04-19

**Authors:** Sergio Rinella, Simona Massimino, Alessia Sorbello, Vincenzo Perciavalle, Marinella Coco

**Affiliations:** ^1^Section of Physiology, Department of Biomedical and Biotechnological Sciences, University of Catania, Catania, Italy; ^2^Department of Sciences of Life, Kore University of Enna, Enna, Italy

**Keywords:** STAI-Y2, testosterone, personological factors, sex-linked behaviors, affective state

## Abstract

This study aimed to identify a possible correlation between the D2:D4 ratio and state and/or trait anxiety in adult healthy subjects and, if so, whether it exists any difference between men and women. In addition, we also wanted to observe whether there is a relationship between participants' age and state and/or trait anxiety. The research involved 125 subjects of both sexes, who were calculated the D2:D4 ratio and were administered the self-assessment questionnaire State-Trait Anxiety Inventory (STAI-Y). Results show that there are positive significant correlations between the D2:D4 ratio and score at state anxiety and trait anxiety, in the total sample. However, if men are examined separately from women, it can be observed that only men have a statistically significant relationship between D2:D4 ratios and state anxiety and trait anxiety. Moreover, about possible relations between the age of participants and state and trait anxiety, a significant negative relationship was observed, without differences between men and women. However, only subjects with a D2:D4 ratio ≥ 1, without differences between men and women, showed a statistically significant negative linear correlation between their age and their state and trait anxiety. The present data allow us to conclude that a low D2:D4 ratio (<1) represents a protective factor against anxiety in both men and women and that this protection seems likely to act throughout life.

## Introduction

Prenatal exposure to sex hormones determines important effects on the development and organization of the brain, which, consequently, will also influence its behavior (Collear and Hines, 1995). Subjects with high levels of testosterone present themselves as more prone to take risks, impulsive, with greater aggressiveness, while subjects with low levels of testosterone present themselves as more prone to reasoning and the study of logical subjects (Manning et al., 2000; Kilduff et al., 2013).

Several markers can detect the effects of prenatal androgens (Cohen-Bendahan et al., 2005), however, among the less invasive measures, the digit ratio allows to estimate prenatal exposure to androgens. It appears detectable in human fetuses as early as 10–40 weeks of gestation and assumes stable levels around 2 years of age (Manning et al., 1998; Manning, 2002a; Malas et al., 2006). It expresses the ratio of the length of the index finger to the ring finger of the right hand (Neyse and Brañas-Garza, 2014) which is measured from the midpoint of the lower crease (where the finger joins the hand) to the tip of the finger (D2:D4).

Generally, the digit ratio shows positive correlations with typical female behaviors and negative correlations with typical male behaviors (Putz et al., 2004). Specifically, it was observed that D2:D4 ratios appear to correlate with certain personological factors such as neuroticism, agreeableness, and openness (Fink et al., 2004, 2006). Moreover, D2:D4 can be a predictive factor of success among high financial brokers (Coates et al., 2009), of success in admission at Italian Medical Schools (Coco et al., 2011), of best performances in sports such as basketball (Tester and Campbell, 2007), skiing (Manning, 2002b), and soccer (Manning and Taylor, 2001), and of aggressive behavior in professional soccer players (Perciavalle et al., 2013) and of the risky decision making (Massimino et al., 2019; Rinella et al., 2019).

Many studies have attempted to verify possible correlations between digit ratio indices and impulsivity, while there are far fewer studies that have explored a possible correlation between the D2:D4 ratio and state and trait anxiety. Usually, the onset of anxiety disorders coincides with puberty, a period when the production of sex hormones is greatest (Yonkers and Kidner, 2002). It is shown that females are more than two times as likely as males to be afflicted with mood disorders (Shear et al., 2000; Pigott, 2003; Kessler et al., 2005; Bekker and van Mens-Verhulst, 2007; Fabio and Caprì, 2019). This disparity has been documented worldwide (Seedat et al., 2009) and would seem to suggest a potential role for gonadal hormones in the onset of anxiety and depressive disorders. In fact, several studies have confirmed that women are more likely to experience mood disorders during puberty, menopause, perimenstrual periods, and postpartum (Ahokas et al., 2001; Parker and Brotchie, 2004; Douma et al., 2005; Solomon and Herman, 2009).

Also, in women, an association is shown between D2:D4 and anxious behavior in childhood (Williams et al., 2003), and also with neuroticism (Fink et al., 2004), considered a precursor to anxiety (Ehrler et al., 1999; Khan et al., 2005).

Moreover, the relationship between testosterone levels, anxiety disorders, and major depressive disorder in humans is evident in males with hypogonadism, who exhibit a significantly higher prevalence of anxiety disorders and major depressive disorder, compared to those with normal physiological levels of androgens (Shores et al., 2004; Zarrouf et al., 2009). Despite a few inconsistent reports, the majority of studies support the case that testosterone yields beneficial effects on mood in men, especially in those with lower than normal levels (McHenry et al., 2014).

Even in the animal world, it has been shown that rhesus monkeys show sexually dimorphic D2:D4, with males having higher ratios than females (Baxter et al., 2017), and male rats deprived of androgens because of perinatal castration display female-typical patterns of anxious behavior (Lucion et al., 1996).

Few studies have used the D2:D4 ratio to explore a possible correlation between state and trait anxiety. Among these, it is possible to mention two. The first one actually identified a correlation between the D2:D4 ratio and anxiety in male university students, females excluded (Evardone and Alexander, 2009). The second instead investigated male subjects suffering from anxiety disorders (de Bruin et al., 2004). The results, whose scores indicated high values of the D2:D4 ratio, allowed us to suppose that these subjects may have been exposed to lower levels of prenatal testosterone. However, in this study female subjects were not assessed. From these observations, we hypothesize that there is a difference between males and females and that a higher digit ratio correlates with an increase in perceived state and trait anxiety.

Therefore, the main purpose of this study was to identify a possible correlation between the D2:D4 ratio and state and/or trait anxiety in adult healthy subjects, and, if so, whether it exists any difference between men and women. In addition, we also wanted to observe whether there is a relationship between participants' age and state and/or trait anxiety.

## Materials and Methods

### Subjects

The experimental sample was composed of 125 healthy adult subjects, of both sexes (66 male and 59 female), of an age group between 20 and 71 years (mean = 31.9; SD = 8.7). Male subjects had a mean age of 33.7 years (±10.1 SD), whereas women had a mean age of 29.9 years (±6.4 SD).

Information was collected on the participant's general condition, health status, and whether they were taking any drugs or substances. Volunteers signed informed consent and were also informed of their right to privacy, non-recognition, and anonymity.

The measurements were performed in agreement with the ethical standards of the Helsinki Declaration and approved by the Ethics Committee Catania 1 (authorization n. 113/2018/PO).

### Procedures

To perform the study, the D2:D4 ratio was measured and a psychological assessment test for measures anxiety was performed (STAI-Y).

- D2:D4 ratio: The participants' right hand was photocopied and the length of the fingers from the metacarpophalangeal crease to the fingertip was measured. It was observed that this fold appears around the 9th week of gestation and is one of the primary folds of the hand (Kimura et al., 1990).
- The D2:D4 ratio has only been determined from the right hand, as right-hand finger ratios show stronger gender differences and appear to be more sensitive to prenatal androgens (Williams et al., 2000; Manning et al., 2007).
- To measure the D2:D4 ratio, we used the practical recommendations suggested by Voracek et al. (2007) and those recently described by Coates and Hebert (2008). In soft tissues, care must be taken to distinguish regular folds from irregular or secondary folds. Irregular folds form later than regular folds, after the 11th week of gestation when the fingers begin to bend, disrupting the dermal surface. Participants' hand prints were measured to determine the D2:D4 ratio by one of the authors using calipers accurate to 0.2 mm.
- State-Trait Anxiety Inventory (Spielberger, 1983): It is composed of 40 items and 2 scales (20 items per scale). On the state scale, participants were asked to describe how they were feeling “right now, that is, at this moment.” Responses were on a 4-point scale ranging from 1 = “Not at all” to 4 = “Very Much So.” On the trait scale, participants were asked to describe how they “generally feel.” Responses were on a 4-point scale ranging from 1 = “Almost Never” to 4 = “Almost Always.” For example, state anxiety items include: “I am tense; I am worried” and “I feel calm; I feel secure.” Trait anxiety items include: “I worry too much over something that really doesn't matter” and “I am content; I am a steady person.” Scores on the 20 items were summed to provide an overall scale score. Higher scores are positively correlated with higher levels of anxiety. Its most current revision is Form Y. Internal consistency coefficients for the scale have ranged from 0.86 to 0.95; test-retest reliability coefficients have ranged from 0.65 to 0.75 over a 2-month interval. Considerable evidence attests to the construct and concurrent validity of the scale (Spielberger, 1989).

### Statistical Analysis

Data were collected and averaged and then compared with the unpaired *t*-test (two-tailed); multiple linear regression and the correlation coefficient of Pearson were also calculated. Significance was set at *p* < 0.05. All descriptive statistics are described as mean _ SD. All analyses were performed by means of GraphPad Prism version 6.00 for Windows (GraphPad Software, La Jolla, California, USA).

## Results

### D2:D4 Ratio

**Figure 1:**
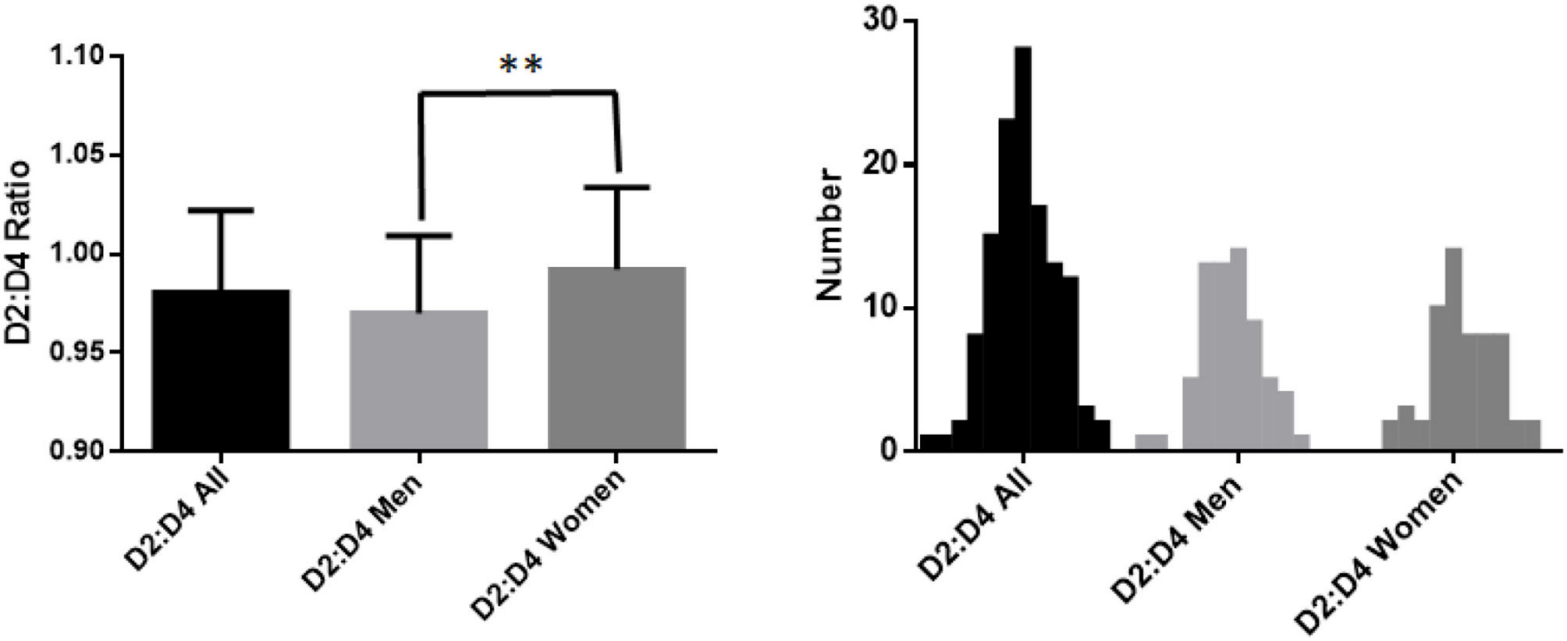
On the left are the mean values of the D2:D4 ratios and on the right is the distribution of the observed values, both of the entire sample, and separately for men and women; **p < 0.01, moderate significant.

Figure 1 shows the values of D2:D4 ratios measured by the participants in the study. On the left of the figure, it can be seen the mean values (±SD) observed in the entire sample, and separately for men and women. Instead, the distributions of observed values for the total sample, and separately for men and women, are shown on the right.

As can be seen, measurement of the D2:D4 ratio in participants of the study showed differences between men and women. In fact, whereas in the entire sample the mean value was 0.98 (±0.041 SD), in men the mean value of the ratio was 0.97 (±0.039 SD) whereas in women it was 0.99 (±0.039 SD).

Comparison of these two latter sets of values using an unpaired *t*-test showed that the difference was statistically significant (*p* = 0.0028).

In contrast, no significant differences were observed in the distribution of observed D2:D4 ratios between men and women.

### State and Trait Anxiety

**Figure 2:**
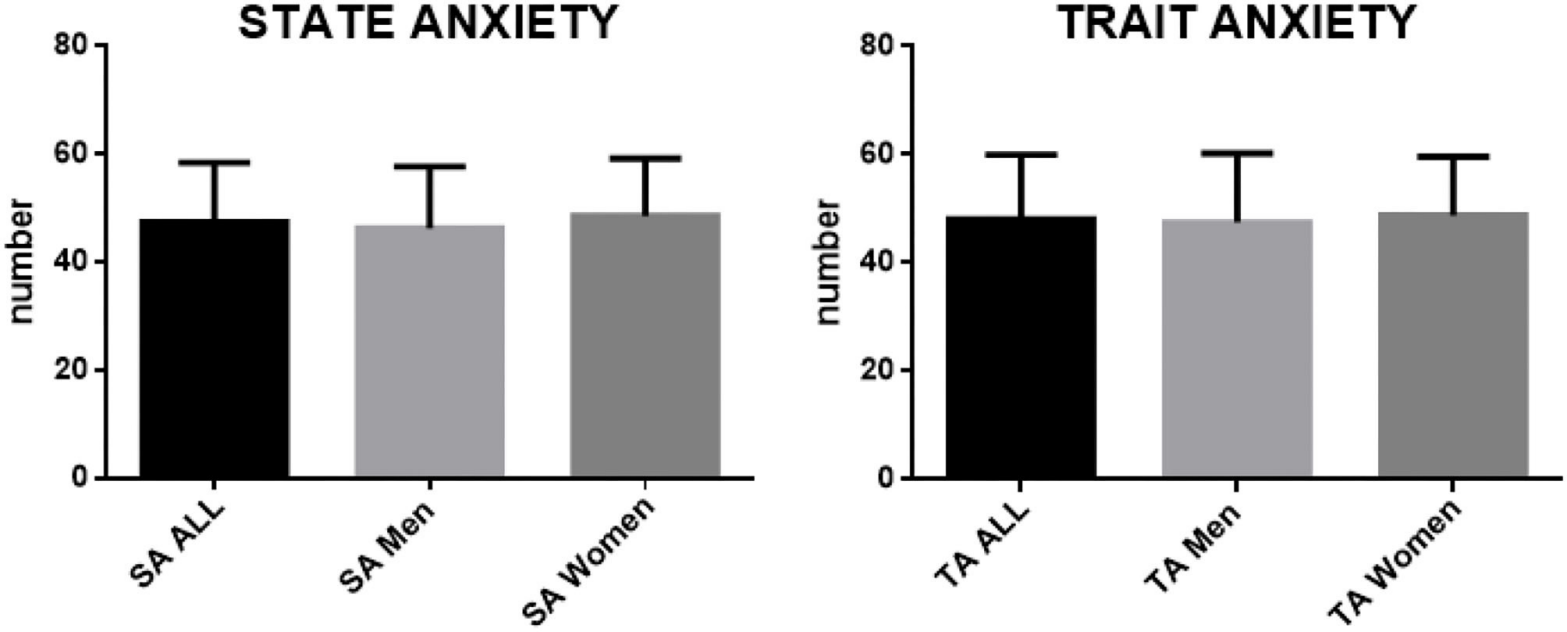
State and trait anxiety values, both of the total sample, and separately for men and women.

Figure 2 shows the values of state anxiety (on the right) and trait anxiety (on the left) measured by the participants in the study. For both state anxiety and trait anxiety, the figure shows the values measured in the entire sample, and separately for men and women.

Concerning the state anxiety, in the entire sample, the mean value was 47.42 (±11.01 SD), in men the mean value was 46.32 (±11.35 SD) whereas in women it was 48.66 (±10.56 SD).

Concerning the trait anxiety, in the whole sample, the mean value was 48.10 (±11.84 SD), in men the mean value was 47.53 (±12.68 SD) whereas in women it was 48.75 (±10.89 SD).

For both state anxiety and trait anxiety, the unpaired *t*-test showed no statistically significant differences between men and women.

### Correlation Between D2:D4 Ratio and Anxiety

**Figure 3:**
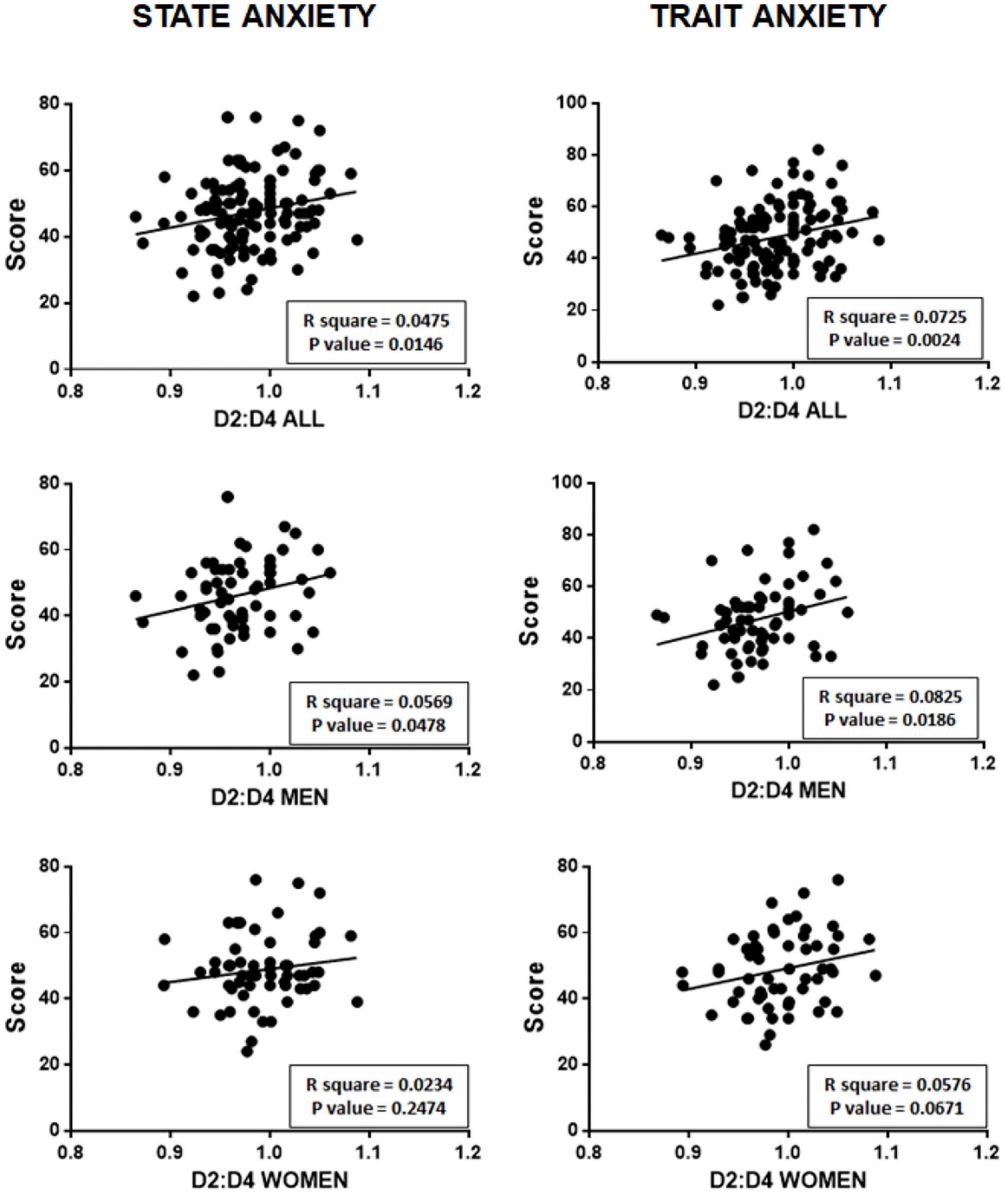
Positive correlations between D2:D4 ratios and state and trait anxiety scores, both of the total sample, and separately for men and women.

Figure 3 shows the correlations between D2:D4 ratios and state anxiety (on the left) and trait anxiety (on the right) at in the entire sample of subjects participating in the study, and separately for men and women.

As can be seen for the entire sample, there is a positive significant relationship between the D2:D4 ratio and score at STAI for both state anxiety (*p* = 0.0146) and trait anxiety (*p* = 0.0024).

However, if men are examined separately from women, it can be observed that only men have a statistically significant relationship between D2:D4 ratios and state anxiety (*p* = 0.0478) and trait anxiety (*P* = 0.0186).

### Correlation Between age and Anxiety

**Figure 4:**
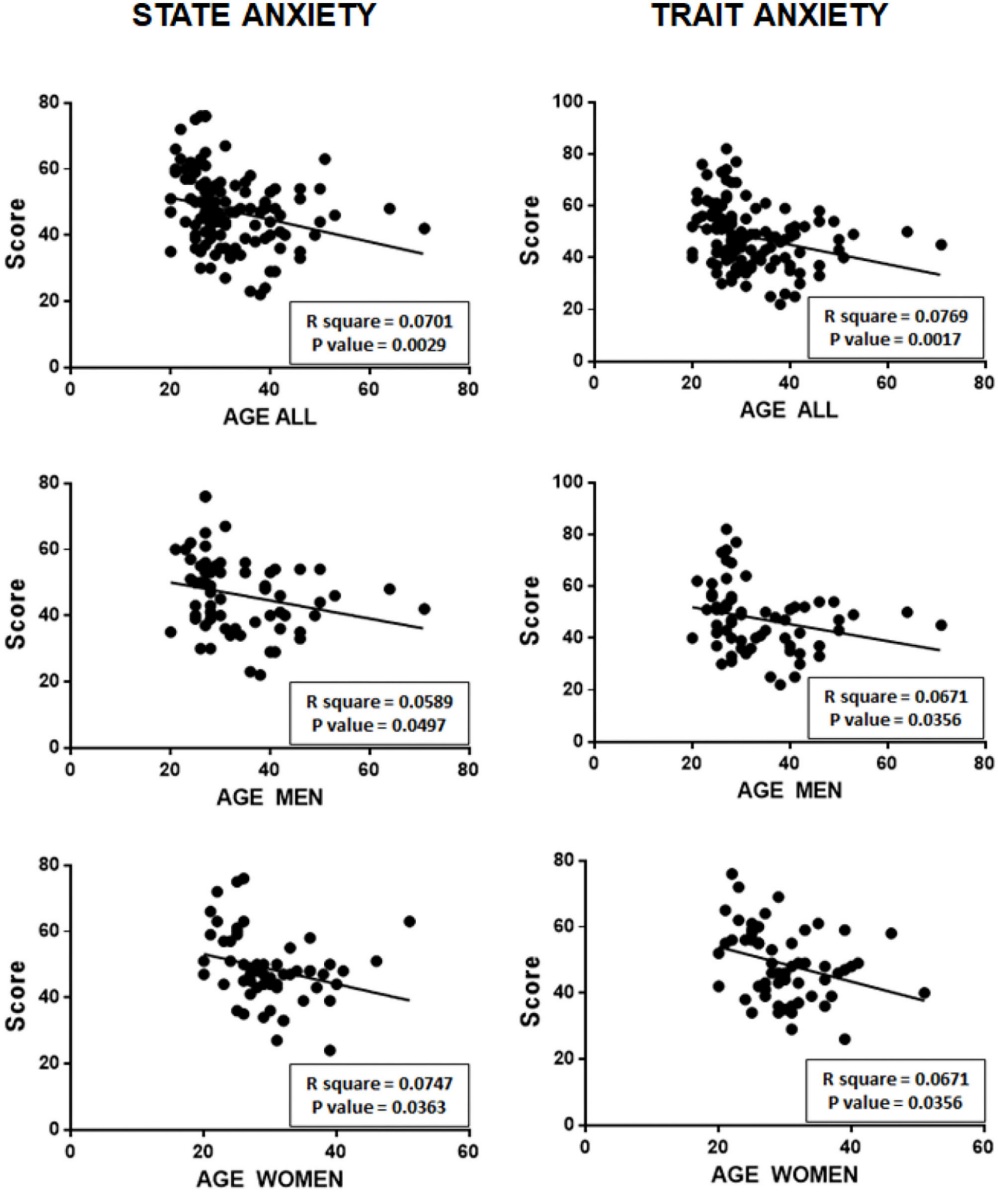
Negative correlations between the ages of participants and state and trait anxiety scores, both of the total sample, and separately for men and women.

Figure 4 shows the correlations between the age of participants and state anxiety (on the left) and trait anxiety (on the right) measured in the entire sample of subjects participating in the study, and separately for men and women.

As can be seen, a significant negative relationship was observed for both state and trait anxiety with age, without differences between men and women.

However, if participants (both the men and women) are separated into individuals with a D2:D4 ratio less than 1 (*N* = 79) and subjects with a D2:D4 ratio equal or greater than 1 (*N* = 46), the situation changes significantly.

**Figure 5:**
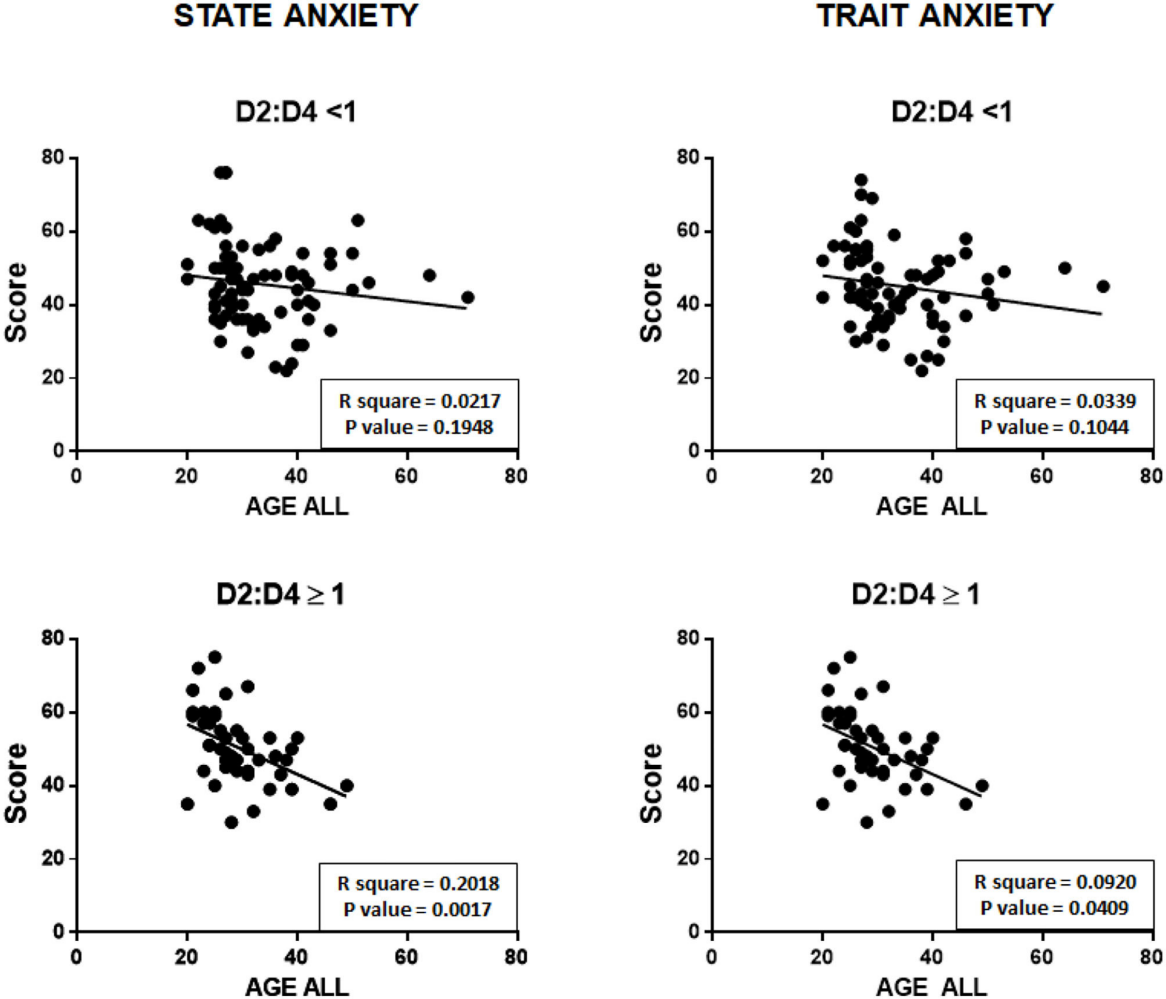
Correlations between the ages of participants and state and trait anxiety scores of the individuals with a D2:D4 ratio ≥ 1.

In fact, as can be seen in Figure 5, individuals with a D2:D4 ratio < 1 did not show a significant correlation between their age and their state anxiety and trait anxiety.

In contrast, individuals with a D2:D4 ratio ≥1 showed a statistically significant negative linear correlation between their age and their state anxiety and trait anxiety.

## Discussion

Anxiety is an uncomfortable feeling, such as worry or fear that can be mild or severe. Everyone experiences anxiety in their life, as for a job interview or an exam or a medical test. Some individuals cope with these situations without great difficulty, while others find it difficult to control their worries. In these individuals, their feelings of anxiety are more constant and can often affect their daily lives.

As said above, the main purpose of this study was to identify a possible correlation between the D2:D4 ratio and state and/or trait anxiety in adult healthy subjects, and, if so, whether it exists any difference between men and women. In addition, we also wanted to observe whether there is a relationship between participants' age and state and/or trait anxiety.

The observed results can be summarized as follows:

- It should first be pointed out that the D2:D4 ratio measured in men was slightly lower (0.97 ± 0.039 SD) than that observed in women (0.99 ± 0.039 SD), and that this difference was statistically significant (*P* = 0.0028).
- Regarding anxiety levels in participants, it should also be noted that, for both state anxiety and trait anxiety, no statistically significant differences were observed between men and women.
- Concerning possible correlations between the D2:D4 ratio and anxiety (summarized in Figure 3), there is a positive significant relationship between the D2:D4 ratio and score at STAI for both state anxiety (*P* = 0.0146) and trait anxiety (*P* = 0.0024). However, if men are examined separately from women, it can be observed that only men have a statistically significant relationship between D2:D4 ratios and state anxiety (*P* = 0.0478) and trait anxiety (*P* = 0.0186).
- About possible relations between the age of participants and state and trait anxiety, a significant negative relationship was observed, without differences between men and women (Figure 4). However, if study participants (both men and women) were separated into individuals with a D2:D4 ratio < 1 (*N* = 79) and with a D2:D4 ratio ≥ 1 (*N* = 46), only subjects with a D2:D4 ratio ≥ 1 showed a statistically significant negative linear correlation between their age and their state and trait anxiety (Figure 5).

## Conclusion

This study confirms what has already been highlighted in the literature (see Manning et al., 2014b), namely, that the D2:D4 ratio is related to the individual's ability to cope with unexpected and/or negative situations, the main cause of anxiety. This ability, although more evident in men, is also detectable in women. This well accords with findings in studies that have emphasized that the D2:D4 ratio tends to be negatively correlated with rates of female workforce participation and that this effect was greatest for the right hand (Manning et al., 2014a).

In summary, the present data allow us to conclude that a low D2:D4 ratio (<1) represents a protective factor against anxiety in both men and women and that this protection seems likely to act throughout life. Certainly, it would be interesting in the future to investigate the correlation of digit ratio with both cognitive performance and psychological protective variables of anxiety, to confer to an anthropometric variable such as digit ratio a predictive value with a social impact.

## Data Availability Statement

The raw data supporting the conclusions of this article will be made available by the authors, without undue reservation.

## Ethics Statement

The studies involving human participants were reviewed and approved by University of Catania. The patients/participants provided their written informed consent to participate in this study. Written informed consent was obtained from the individual(s) for the publication of any potentially identifiable images or data included in this article.

## Author Contributions

SR, SM, MC, and VP contributed to the conception and design of the study. SR and SM were responsible for data collection and VP for statistical analysis. All the authors were responsible for the drafting and finalization of the manuscript, contributed to manuscript revision, and approved the submitted version of the manuscript.

## Conflict of Interest

The authors declare that the research was conducted in the absence of any commercial or financial relationships that could be construed as a potential conflict of interest.

## Publisher's Note

All claims expressed in this article are solely those of the authors and do not necessarily represent those of their affiliated organizations, or those of the publisher, the editors and the reviewers. Any product that may be evaluated in this article, or claim that may be made by its manufacturer, is not guaranteed or endorsed by the publisher.
